# Relationship between nontraditional lipid parameters and the risk of type 2 diabetes in individuals recovered from dyslipidemia: a cohort study

**DOI:** 10.3389/fendo.2025.1610091

**Published:** 2025-09-25

**Authors:** Yuxian Wang, Zekai Chen, Zhen He, Hailun Qin, Kuangyi Wu, Huancong Zheng, Peng Fu, Zefeng Cai, Weiqiang Wu, Yulong Lan, Dan Wu, Shouling Wu, Youren Chen

**Affiliations:** ^1^ Department of Cardiology, Second Affiliated Hospital of Shantou University Medical College, Shantou, Guangdong, China; ^2^ Department of Epidemiology, University Medical Center Groningen, University of Groningen, Groningen, Netherlands; ^3^ Department of Cardiology, University Medical Center Groningen, University of Groningen, Groningen, Netherlands; ^4^ Centre for Precision Health, Edith Cowan University School of Medical and Health Sciences, Joondalup, WA, Australia; ^5^ Department of Cardiology, Kailuan General Hospital, Tangshan, China

**Keywords:** diabetes, cohort, epidemiology, nontraditional lipid parameters, lipids

## Abstract

**Aims/Introduction:**

Lipid profiles are essential for assessing type 2 diabetes (T2D) risk, but individuals who recover from dyslipidemia are often overlooked, leaving their residual risk unclear. This study aimed to evaluate T2D risk in individuals with varying lipid change patterns and investigate the associations between traditional and novel lipid parameters with T2D risk among those recovered from dyslipidemia.

**Materials and Methods:**

In this prospective cohort study of the Kailuan Study, 39,283 non-diabetic participants were followed to evaluate T2D risk across lipid change patterns using the Cox proportional hazards models. A subset of 3,850 individuals recovered from dyslipidemia was analyzed to examine the associations between both traditional and novel lipid parameters and T2D risk using Cox models and restricted cubic splines. Predictive performance was assessed using the C-index.

**Results:**

During follow-up, 5,223 participants developed T2D. Individuals recovered from dyslipidemia had a significantly higher T2D risk (hazard ratio [HR], 1.37; 95% CI, 1.25-1.51) compared to those with persistent normal lipid levels. In this group, high-density lipoprotein cholesterol (HDL-C) was inversely associated with T2D risk, while triglyceride (TG), lipoprotein combine index (LCI), atherogenic index of plasma (AIP), non-HDL-C, Castelli’s index-I, Castelli’s index-II and triglyceride-glucose index (TyG) were positively associated. AIP and TyG outperformed other parameters in predictive ability.

**Conclusions:**

Individuals recovered from dyslipidemia remain at an elevated risk for T2D. Novel lipid parameters, particularly AIP and TyG, demonstrate superior predictive performance in this group, providing valuable insights for risk stratification and targeted prevention strategies.

## Introduction

Diabetes is a leading global health concern, affecting 537 million adults in 2021, with this number projected to 783 million by 2045. That same year, diabetes was responsible for 6.7 million deaths and healthcare expenditures of 966 billion United States Dollars, imposing a significant strain on both public health and global economy (1).

Dyslipidemia is closely associated with type 2 diabetes (T2D). Lipid metabolism disorders play a significant role in T2D pathophysiology (2–4). Conversely, T2D patients often exhibit lipoprotein abnormalities, including elevated triglycerides (TG) and low-density lipoprotein cholesterol (LDL-C), alongside reduced high-density lipoprotein cholesterol (HDL-C) levels (5, 6). Traditional lipid parameters, as well as composite nontraditional lipids indices such as the atherogenic index of plasma (AIP) and the triglyceride-glucose index (TyG), provide valuable tools for diabetes risk assessment (7, 8). Compared with hyperinsulinemic-euglycemic clamp test, the gold standard for evaluating insulin resistance (9), lipid parameters are cost-effective, simple, and well-suited for large-scale screenings.

However, the long-term effects of lipid metabolism disorders may persist even after recovery, leading to lasting damage to pancreatic β-cells (10) and the kidneys (11). This raise concerns that individuals recovered from dyslipidemia may remain at elevated T2D. Current guidelines, however, do not address whether such individuals face continued risk. Additionally, the role of lipid parameters—both traditional and nontraditional—in predicting T2D risk in this specific group remains unclear. This study aims to investigate whether individuals who have recovered from dyslipidemia still face an elevated risk of developing T2D. Furthermore, it evaluates the association and predictive value of both traditional and nontraditional lipid parameters for T2D in this specific population.

## Methods

### Study population

**Figure 1:**
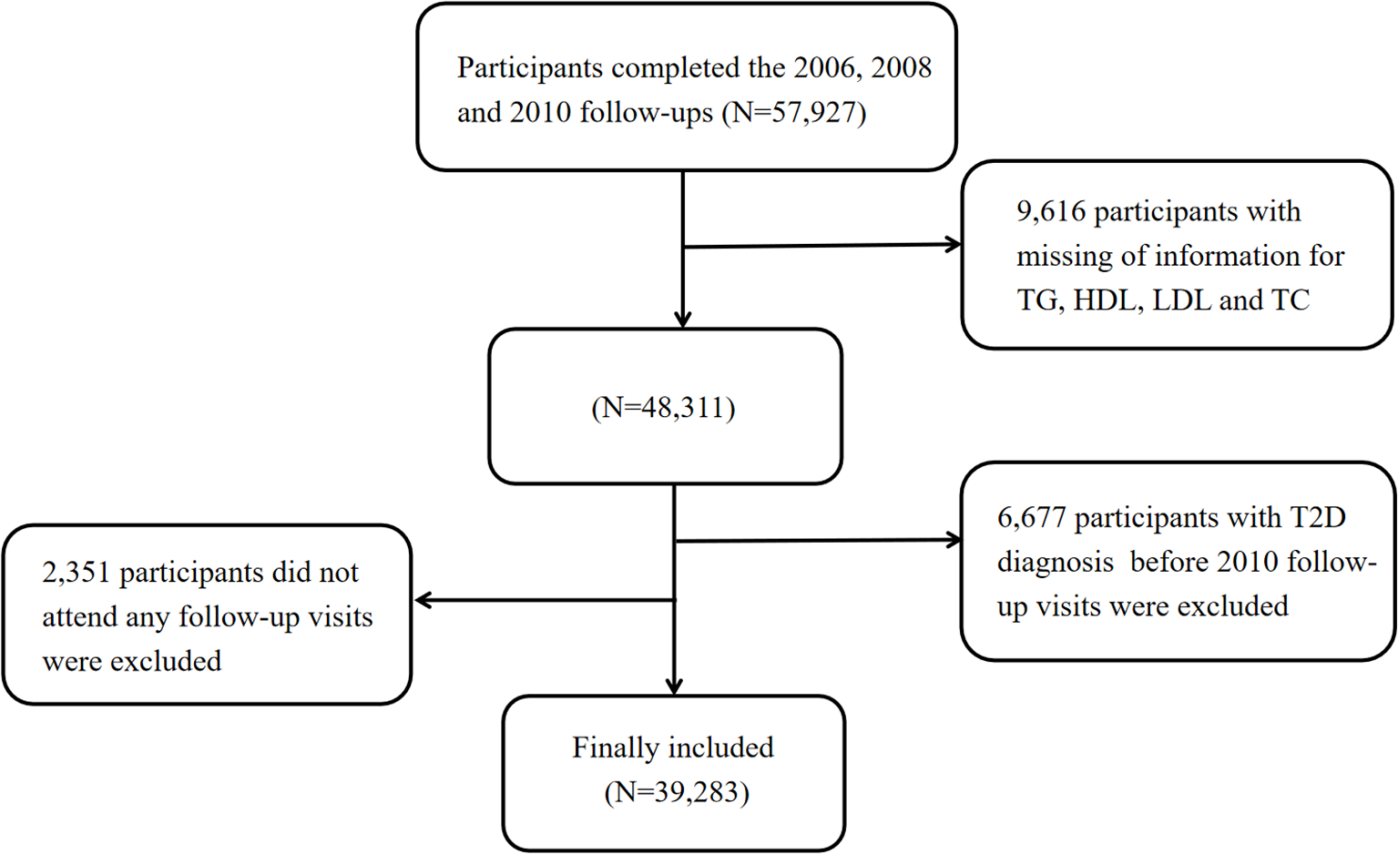
Flowchart. T2D, Type 2 Diabetes; HDL, High-Density Lipoprotein; LDL, Low-Density Lipoprotein; TG, Triglycerides; TC, Total Cholesterol.

The Kailuan Study is a prospective cohort study involving employees and retirees from the Kailuan Group in Tangshan City, China. During the baseline survey (2006-2007), over 100,000 participants were recruited, with biennial follow-ups conducted thereafter. Details of the study design and procedures have been specified elsewhere (12, 13). In this analysis, we included participants who completed health check-ups in 2006, 2008, and 2010 (n= 57,927). Exclusion criteria were as follows: participants with missing data on dyslipidemia diagnosis (TG, HDL-C, LDL-C, and total cholesterol (TC), n=9,616); those diagnosed with T2D at or before the 2010 survey (n = 6,677); and individuals who did not attend any follow-up visits (n = 2,351). Ultimately, 39,283 participants were included in the study (**Figure 1**). The Kailuan Study was conducted in accordance with the Declaration of Helsinki, approved by the ethics committee of Kailuan General Hospital, with written informed consent from all participants.

### Study design overview

In the first step, 39,283 non-diabetic participants were divided into four groups based on their dyslipidemia progression to assess the risk of T2D associated with different lipid change patterns using the Cox proportional hazards model. In the second step, we focused on individuals who had recovered from dyslipidemia (n=3,850), employing the Cox proportional hazards model and restricted cubic splines (RCS) to explore the association between traditional and nontraditional lipid parameters and diabetes risk. The predictive performance of these parameters was estimated using the C-index.

### Definitions

#### Definition of dyslipidemia

Dyslipidemia was defined as having at least one of the following: increased TC (≥6.20 mmol/L), LDL-C (>4.13 mmol/L), TG (>2.25 mmol/L), decreased HDL-C (<1.03 mmol/L) (14), or current use of lipid-lowering medication.

#### Definition of dyslipidemia evolution groupings

1. Persistent Normal: No diagnosis and no self-reported history of dyslipidemia in 2006 physical examination, and no diagnosis in 2008 and 2010.
2. Dyslipidemia-Recovered (individuals recovered from dyslipidemia): Diagnosed or self-reported history of dyslipidemia in 2006 physical examination, but no diagnosis in 2008 and 2010.
3. Normal-Dyslipidemia: No diagnosis and no self-reported history of dyslipidemia in 2006 physical examination, but diagnosed in 2008 or 2010.
4. Persistent Dyslipidemia: Diagnosed or self-reported history of dyslipidemia in 2006 physical examination, and diagnosed in 2008 or 2010.

### Definition of nontraditional lipid parameters

Lipoprotein combine index (LCI)= TC×TG×LDL-C/HDL-C (7);
AIP= lg (TG/HDL-C) (15);
Non-high density lipoprotein (NHDL) =TC−HDL-C (16);
Castelli’s index-I (CRI-I) =TC/HDL-C (17);
Castelli’s index-II (CRI-II) =LDL-C/HDL-C (17);
Remnant cholesterol (RC) =TC−HDL-C−LDL-C (18);
RC/HDL-C ratio (RHC) =RC/HDL-C (19);
TyG=ln [TG (mg/dL) × fasting blood glucose (FBG) (mg/dL)/2] (20).

#### Assessment of exposure and covariates

Since 2006, biennial follow-up surveys were conducted by trained staff. These included standardized face-to-face interviews to collect sociodemographic information (age, sex, education level), lifestyle factors (smoking, alcohol consumption, physical activity), and antihypertensive medication history. Height and weight were measured to calculate body mass index (BMI) as weight (kg) divided by height squared (m²). Education level was categorized into three groups: primary school or below, middle to high school, and above high school. Smoking and alcohol consumption were classified as never or ever, while physical activity was categorized as ≤3 times per week or >3 times per week, with each session lasting at least 20 minutes. Blood pressure was measured three times in an upright seated position after at least 5 minutes of rest, with the mean value recorded. Fasting blood samples were collected for the analysis of serum creatinine, TG, TC, LDL-C, and HDL-C, performing using a Hitachi 747 automated analyzer. Estimated glomerular filtration rate (eGFR) was calculated from serum creatinine levels according to the Chronic Kidney Disease Epidemiology Collaboration equation (21).

### Assessment of outcomes

The assessment period began at the 2010 physical examination (baseline) and ended upon the first occurrence of T2D, death, or the study’s conclusion on December 31, 2022. New-onset T2D was defined as either FBG ≥7.0 mmol/L, self-report of a physician diagnosis, or self-report current use of antidiabetic medication (22) during the follow-up period. FBG levels were measured using Hitachi 747 automated analyzer.

### Statistical analysis

Normally distributed continuous variables were described using the mean ± standard deviation and compared between groups using one-way analysis of variance. Non-normally distributed continuous variables were presented as median and interquartile range and compared between groups using the Kruskal-Wallis test. Categorical variables were expressed as numbers with percentages, with differences between groups assessed using the chi-square test, with 2-sided P values <0.05 considered statistically significant.

#### Step 1: lipid change patterns and T2D Risk

Using the persistent normal group as the reference, hazard ratios (HRs) and 95% confidence intervals (CIs) for the association between lipid change patterns and the risk of T2D were estimated using Cox proportional hazards models. Model 1 adjusted for age and sex, while Model 2 further adjusted for baseline systolic blood pressure (SBP), eGFR, BMI, smoking, alcohol consumption, physical activity, education level, and antihypertensive medication use.

Stratified analyses and interaction tests were conducted to evaluate potential modifications of the association between lipid change patterns and T2D across different subgroups, including age (<60 or ≥60 years), sex (men or women) and BMI (≥24 or <24 kg/m²).

A series of sensitivity analyses were performed to assess the robustness of the results. First, a lagged analysis was conducted by excluding the first two years of follow-up to reduce the possibility of reverse causation. Second, competing risk regression (Fine-Gray model) was used to address potential mortality-related confounding. In the third and fourth sensitivity analyses, participants receiving antihypertensive or lipid-lowering treatment at baseline were excluded. In the final sensitivity analysis, we adjusted for baseline FBG.

#### Step 2: lipid parameters and T2D risk in dyslipidemia-recovered individuals

Cox proportional hazards models assessed the association between various lipid parameters and T2D. Lipid parameters were analyzed both as quartiles and as continuous variables, with adjustment in Model 1 and Model 2 consistent with those used in Step 1. Multivariate RCS regression was used to evaluate the nonlinear relationships between the lipid parameters and T2D, with adjustments as described in Model 2 above. The RCS model included three knots located at 25th, 50^th^, and 75th percentile, with the median values of each parameter were set as reference points. To assess the predictive value of lipid parameters, we utilized a univariable model to calculate the C-index as an indicator of predictive performance. Stratified analysis was conducted for individuals with or without prediabetes (5.6mmol/L≤FBG<7.0mmol/L) (23) to evaluate differences in the association between lipid parameters and T2D.

In this study, the proportional hazards assumption was evaluated using Schoenfeld residuals, and results indicated that all models met the PH assumption (P > 0.05). Missing values in the covariates (**Supplementary Table S1**) included in the Cox regression analysis were imputed using multiple imputation by chained equations.

## Results

### Baseline characteristics of participants

Among the 39,283 participants, 77.7% were men, and the average age of the total population was 51.9 ± 11.7 years. Baseline characteristics are presented in **Table 1**. Compared to the persistent normal group and the dyslipidemia-recovered group, individuals in the normal-developed group and the persistent dyslipidemia group were more likely to be male, exhibited higher BMI, SBP and FBG levels, and had greater proportions of smokers, alcohol users, and individuals with lower education levels. In addition, compared with the persistent normal group, the dyslipidemia-recovered group exhibited higher TC, LDL-C, TG, SBP, BMI, and FBG, lower HDL-C and eGFR, as well as higher prevalence of ever smoking and ever drinking, and lower education attainment and physical activity. Neither the persistent normal group nor the dyslipidemia-recovered group reported the use of lipid-lowering medications at baseline.

**Table 1:** Baseline characteristics (N = 39,283).

Variables	Total	Persistent normal	Dyslipidemia- recovered	Normal- developed	Persistent dyslipidemia	P value
Participants, N (%)	39,283	18,209 (46.4)	3,850 (9.8)	8,803 (22.4)	8,421 (21.4)	/
Age, years	51.9 ± 11.7	51.6 ± 12.0	52.8 ± 11.5	51.0 ± 11.6	53.0 ± 11.0	<0.001
Male, N (%)	30,506 (77.7)	13,546 (74.4)	3,134 (81.4)	6,976 (79.2)	6,850 (81.3)	<0.001
Body mass index, kg/m2	25.0 ± 3.3	24.3 ± 3.2	25.0 ± 3.3	25.4 ± 3.3	26.1 ± 3.2	<0.001
Systolic blood pressure, mm Hg	129.5 ± 18.7	127.3 ± 18.8	130.7 ± 18.8	129.3 ± 18.2	133.7 ± 18.4	<0.001
eGFR, ml/min/1.73m2	91.0 ± 18.8	90.7 ± 19.3	87.2 ± 19.0	93.1 ± 18.1	91.0 ± 18.0	<0.001
Triglycerides, mmol/L	1.3 (0.9-1.9)	1.0 (0.8-1.4)	1.2 (0.9-1.5)	1.6 (1.1-2.4)	2.3 (1.4-3.3)	<0.001
Total cholesterol, mmol/L	5.1 ± 1.2	4.7 ± 0.7	4.9 ± 0.7	5.2 ± 1.7	5.6 ± 1.6	<0.001
High-density lipoprotein, mmol/L	1.5 ± 0.4	1.6 ± 0.4	1.5 ± 0.4	1.5 ± 0.5	1.4 ± 0.5	<0.001
Low-density lipoprotein, mmol/L	2.6 ± 0.8	2.4 ± 0.6	2.5 ± 0.7	2.7 ± 0.8	2.8 ± 1.0	<0.001
Fasting blood glucose, mmol/L	5.3 ± 0.6	5.2 ± 0.6	5.3 ± 0.6	5.3 ± 0.6	5.4 ± 0.6	<0.001
Ever smoker, N (%)	15,163 (38.6)	6,280 (34.5)	1,456 (37.8)	3,617 (41.1)	3,810 (45.2)	<0.001
Ever drinker, N (%)	14,141 (36.0)	5,814 (31.9)	1,348 (35.0)	3,431 (39.0)	3,548 (42.1)	<0.001
Education, below middle school, N (%)	2,508 (6.4)	1,078 (5.9)	250 (6.5)	585 (6.7)	595 (7.1)	<0.001
Education, middle/high school N (%)	31,983 (81.4)	14,760 (81.1)	3,262 (84.7)	7,045 (80.0)	6,916 (82.1)	
Education, above high school, N (%)	4,792 (12.2)	2,371 (13.0)	338 (8.8)	1,173 (13.3)	910 (11.0)	
Active physical activity, N (%)	5,312 (13.5)	2,331 (12.8)	477 (12.4)	1,250 (14.2)	1,254 (15.0)	<0.001
Antihypertensive drugs, N (%)	4,169 (10.6)	1,345 (7.4)	388 (10.1)	989 (11.2)	1,447 (17.2)	<0.001
Lipid-lowering drugs, N (%)	323 (0.8)	0 (0)	0 (0)	118 (1.3)	205 (2.4)	<0.001

Data are presented as mean ± SD, median (interquartile range), or number (percentage). eGFR, estimated glomerular filtration rate.

### Relationship between dyslipidemia evolution and diabetes

**Figure 2:**
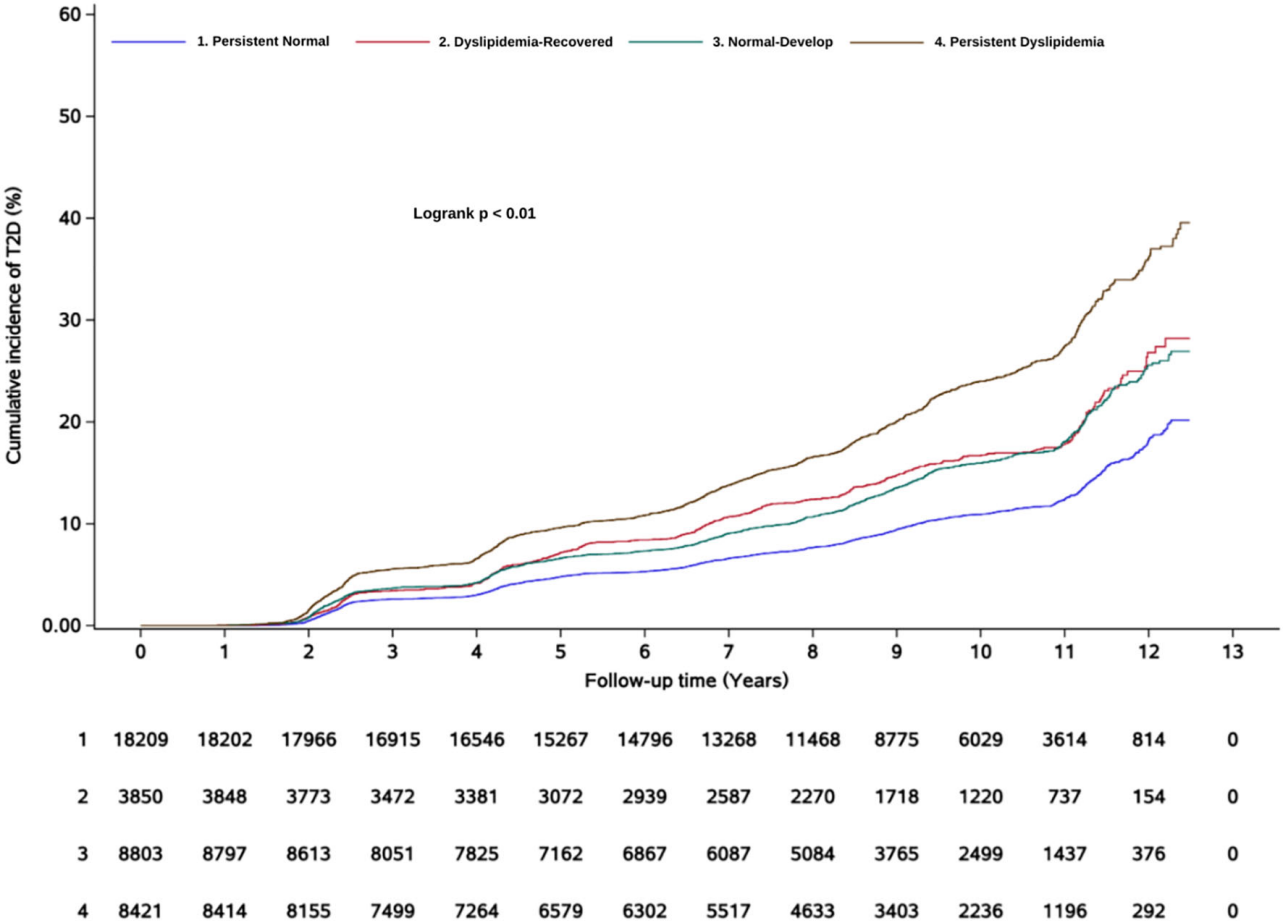
Kaplan–Meier incidence rate of diabetes by dyslipidemia evolution.

After an average follow-up duration of 8.8 (interquartile range: 6.6-10.4) years, a total of 5,223 subjects developed new-onset T2D. The cumulative incidence of T2D is shown in **Figure 2**. In Model 1, compared with the persistent normal group, the dyslipidemia-recovered group (HR = 1.52, 95% CI: 1.38–1.67), the normal-developed group (HR = 1.47, 95% CI: 1.37–1.58), and the persistent dyslipidemia group (HR = 2.23, 95% CI: 2.08–2.38) showed 52%, 47%, and 123% higher risks of T2D, respectively. In the fully adjusted model, relative to the persistent normal group, the risk of T2D remained significantly higher in the dyslipidemia-recovered group (HR = 1.37, 95% CI: 1.25–1.51), the normal-developed group (HR = 1.32, 95% CI: 1.22–1.42), and the persistent dyslipidemia group (HR = 1.78, 95% CI: 1.66–1.91), although the effect sizes were attenuated (**Table 2**).

**Table 2:** HRs (95% CIs) for diabetes according to dyslipidemia evolution.

Metrics	Persistent normal	Dyslipidemia- recovered	Normal- developed	Persistent dyslipidemia	P value
Events/Total	1,780/18,209	559/3,850	1,187/8,803	1,697/8,421	
IR, per 1000 person-years	11.69	18.03	16.77	25.91	
Model 1	1 (reference)	1.52 (1.38-1.67)	1.47 (1.37-1.58)	2.23 (2.08-2.38)	<0.001
Model 2	1 (reference)	1.37 (1.25-1.51)	1.32 (1.22-1.42)	1.78 (1.66-1.91)	<0.001

Model 1 was adjusted for age and sex.

Model 2 was further adjusted for systolic blood pressure, estimated glomerular filtration rate, body mass index, smoking, alcohol consumption, physical activity, education level, and the use of antihypertensive medications.

HR, hazard ratio; IR, incidence rate.

### Stratified analyses and sensitivity analyses

The results were consistent with the primary findings across different age groups, for both men and women, and across BMI categories. In all subgroups, individuals recovered from a past dyslipidemia had a significantly increased risk of developing T2D compared to those with healthy lipid levels, and in younger individuals, males, and those who are overweight, the risk was also significantly higher than that observed in individuals with newly developed dyslipidemia (**Table 3**).

**Table 3:** Stratified analysis.

Metrics	Persistent normal	Dyslipidemia-recovered	Normal-developed	Persistent dyslipidemia	P value	P for interaction
Age						0.812
< 60 years	n=14,040	n=2,880	n=7,069	n=6,340		
Events/Total	1,336/14,040	417/2,880	941/7,069	1,302/6,340		
IR, per 1000 person-years	11.05	17.45	16.12	25.82		
Model	1 (reference)	1.36 (1.22-1.52)	1.29 (1.18-1.40)	1.77 (1.64-1.92)	<0.001	
≥ 60 years	n=4,169	n=970	n=1,734	n=2,081		
Events/Total	444/4,169	142/970	246/1,734	395/2,081		
IR, per 1000 person-years	14.12	19.98	19.83	26.21		
Model	1 (reference)	1.35 (1.12-1.63)	1.37 (1.17-1.61)	1.67 (1.45-1.92)	0.003	
Gender						<0.001
Male	n=13,546	n=3,134	n=6,976	n=6,850		
Events/Total	1,417/13,546	473/3,134	968/6,976	1,371/6,850		
IR, per 1000 person-years	12.66	18.89	17.34	25.75		
Model	1 (reference)	1.36 (1.23-1.51)	1.28 (1.17-1.39)	1.68 (1.56-1.82)	<0.001	
Female	n=4,663	n=716	n=1,827	n=1,571		
Events/Total	363/4,663	86/716	219/1,827	326/1,571		
IR, per 1000 person-years	8.99	14.43	14.65	26.59		
Model	1 (reference)	1.34 (1.05-1.69)	1.44 (1.21-1.70)	2.16 (1.84-2.53)	<0.001	
BMI						<0.001
≥ 24 kg/m2	n=9,450	n=2,312	n=5,798	n=6,277		
Events/Total	1,260/9,450	411/2,312	924/5,798	1,411/6,277		
IR, per 1000 person-years	16.17	22.29	20	29.04		
Model	1 (reference)	1.30 (1.16-1.45)	1.23 (1.13-1.34)	1.65 (1.53-1.79)	<0.001	
< 24 kg/m2	n=8,759	n=1,538	n=3,005	n=2,144		
Events/Total	520/8,759	148/1,538	263/3,005	286/2,144		
IR, per 1000 person-years	6.99	11.78	10.71	16.91		
Model	1 (reference)	1.53 (1.27-1.84)	1.54 (1.32-1.78)	2.21 (1.91-2.57)	0.001	

Model was adjusted for age, sex, systolic blood pressure, estimated glomerular filtration rate, body mass index, smoking, alcohol consumption, physical activity, education level, and the use of antihypertensive medications.

HR, hazard ratio; IR, incidence rate; BMI, body mass index.

In the sensitivity analyses (**Supplementary Tables S2**-**S6**), the results from the 2-year lagged analysis and competing risk regression were consistent with the primary analysis. The findings remained robust after excluding participants who were taking antihypertensive or lipid-lowering medications. Similarly, the results remained robust after adjusting for baseline FBG.

### Relationship between lipid parameters and T2D in individuals with a history of dyslipidemia

**Figure 3:**
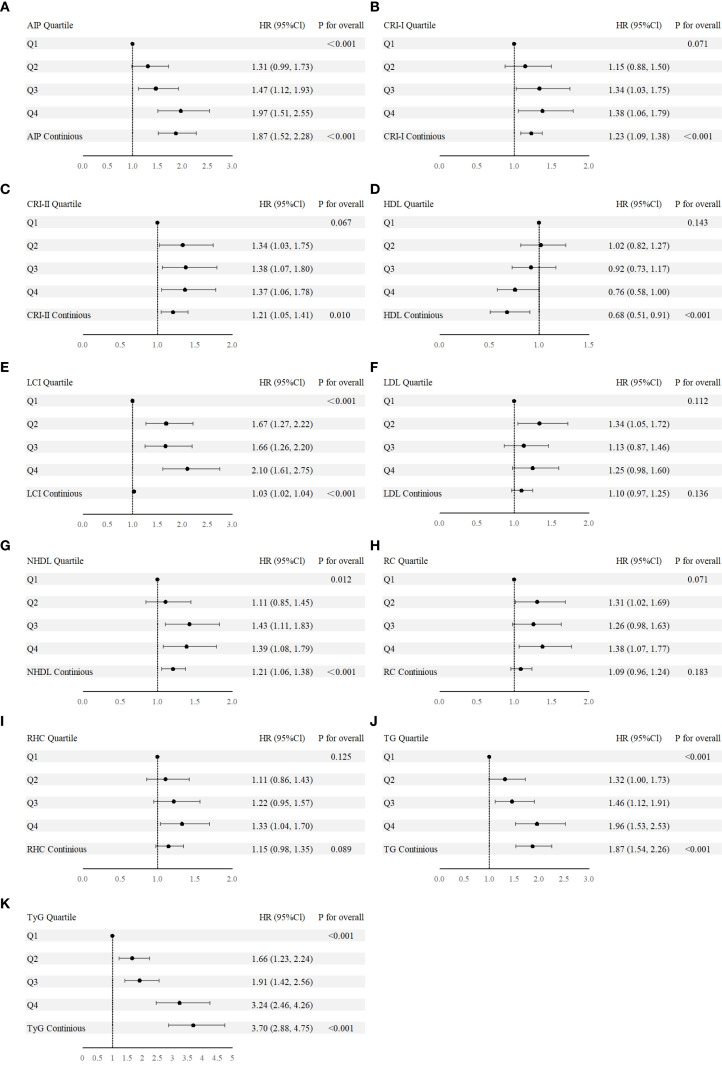
**(A-K)** Forest plot of nontraditional and traditional lipid parameters presented as continuous variables and quartile from Cox regression analysis. AIP, Atherogenic Index of Plasma; Cl, confidence interval; CRI-I, Castelli’s Index-I; CRI-II, Castelli’s Index-II; HDL, High Density Lipoprotein; HR, Hazard Ratio; LCI, Lipoprotein Combine Index; LDL, Low Density Lipoprotein; NHDL, Non-HDL; RC, Remnant Cholesterol; RHC, RC/HDL Ratio; TG, Triglyceride; TyG, Triglyceride-Glucose Index.

**Figure 4:**
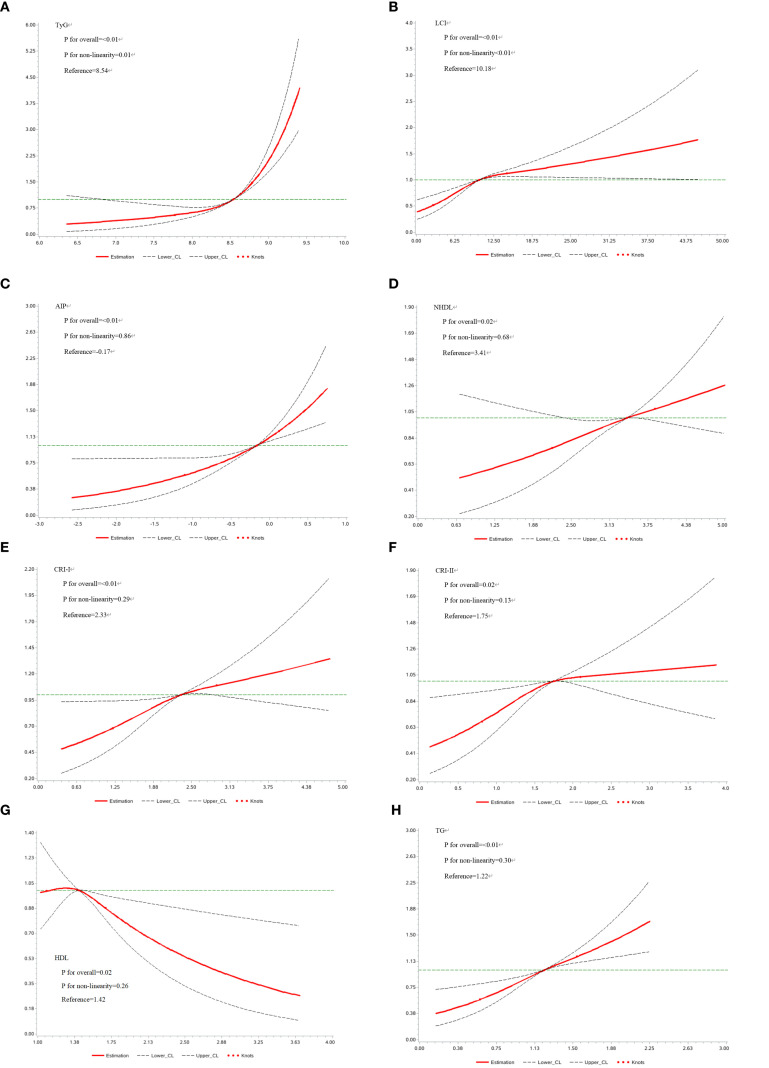
**(A-H)** Restricted Cubic Spline analysis of lipid parameters. AIP, Atherogenic Index of Plasma; CRI-I, Castelli’s Index-I; CRI-II, Castelli’s Index-II; HDL, High Density Lipoprotein; LCI, Lipoprotein Combine Index; LDL, Low Density Lipoprotein; NHDL, Non-HDL; RC, Remnant Cholesterol; RHC, RC/HDL Ratio; TG, Triglyceride; TyG, Triglyceride-Glucose Index.

The second step focused on 3,850 individuals recovered from dyslipidemia and examined the association between both traditional and nontraditional lipid parameters and T2D. In continuous variable analysis, HDL-C was found to be negatively associated with T2D risk (HR = 0.68, 95% CI: 0.51, 0.91). In contrast, positive associations with T2D risk were observed for TG (HR = 1.87, 95% CI: 1.54, 2.26), LCI (HR = 1.03, 95% CI: 1.02, 1.04), AIP (HR = 1.87, 95% CI: 1.52, 2.28), NHDL (HR = 1.21, 95% CI: 1.06, 1.38), Castelli’s index-I (HR = 1.23, 95% CI: 1.09, 1.38), Castelli’s index-II (HR = 1.21, 95% CI: 1.05, 1.41), and TyG (HR = 3.70, 95% CI: 2.88, 4.75). When categorized into quartiles, LCI, AIP, NHDL, TyG, and TG remained significantly associated with increased T2D risk (**Figures 3A-K**). The complete results and the quartile cut-off values for lipid parameters are shown in **Supplementary Tables S7**, **S8**, respectively. Additionally, RCS analysis was used to explore potential nonlinear relationships among lipid parameters. TyG and LCI demonstrated nonlinear associations, while the other parameters showed linear associations (**Figures 4A-H**).

**Figure 5:**
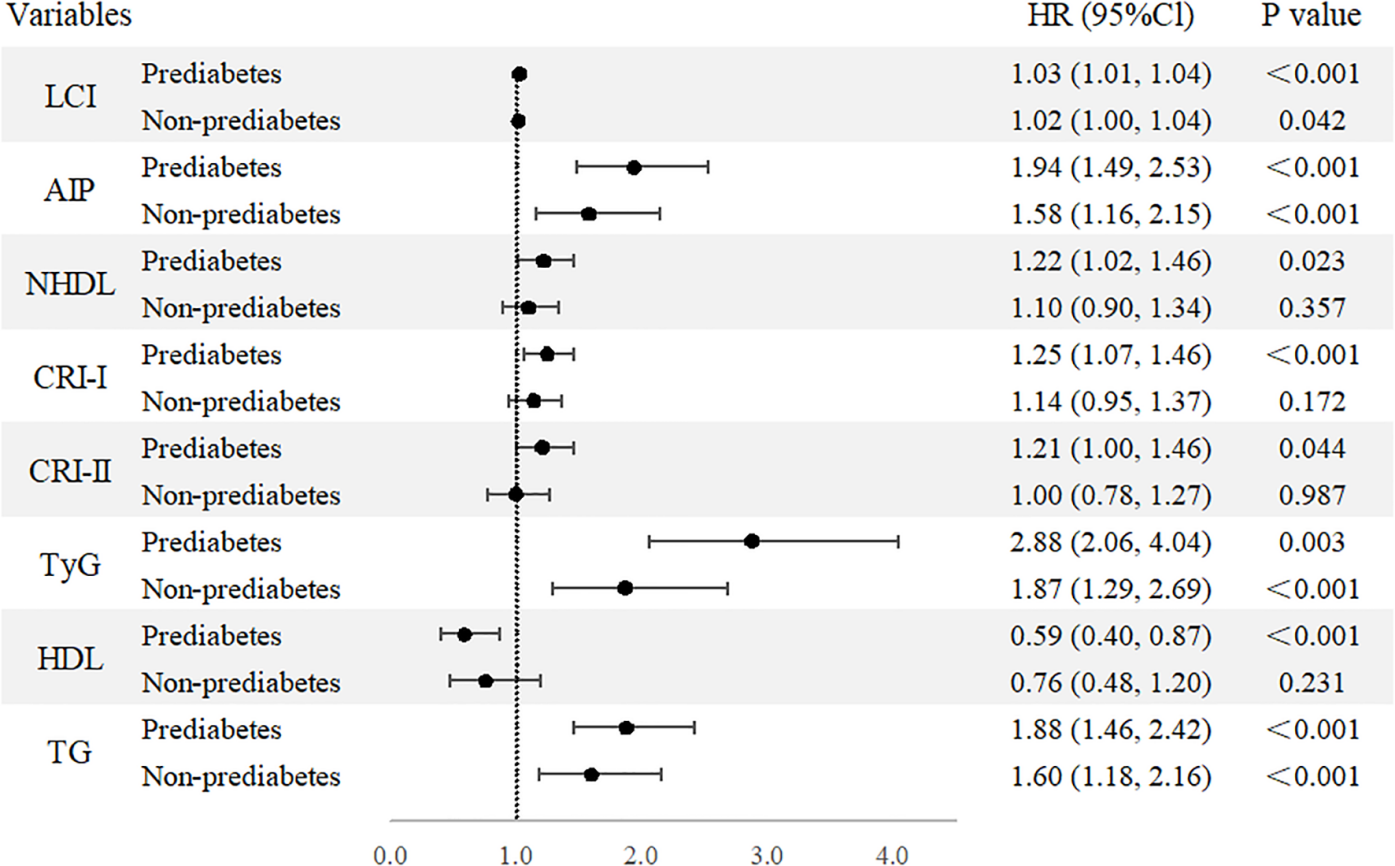
Forest plot of stratified analysis based on pre-diabetes status. Forest plot of nontraditional and traditional lipid parameters presented as continuous variables and quartile from Cox regression analysis. AIP, Atherogenic Index of Plasma; Cl, confidence interval; CRI-I, Castelli’s Index-I; CRI-II, Castelli’s Index-II; HDL, High Density Lipoprotein; HR, Hazard Ratio; LCI, Lipoprotein Combine Index; LDL, Low Density Lipoprotein; NHDL, Non-HDL; RC, Remnant Cholesterol; RHC, RC/HDL Ratio; TG, Triglyceride; TyG, Triglyceride-Glucose Index.

We conducted a stratified analysis focusing on parameters that were significantly associated with T2D in prior analyses. In this analysis, all selected parameters demonstrated significant associations within the prediabetes group, while only LCI, AIP, TyG, and TG remained consistently associated in the non-prediabetes group (**Figure 5**). The results for each 1-standard deviation change in lipid parameters are presented in **Supplementary Table S9**.

### Predictive values of lipid parameters and T2D

For traditional lipid parameters and nontraditional lipid parameters which were previously found to be significantly associated with T2D, we compared their accuracy in identifying T2D using C-index of the univariable models (**Supplementary Table S10**). Among both traditional and nontraditional lipid parameters, TyG showed the strongest ability to identify both overall populations with a C-index of 0.65 (95% CI: 0.62, 0.67) and prediabetic populations with a C-index of 0.62 (95% CI: 0.58, 0.65), while AIP had the best C-index (0.58, 95% CI: 0.55, 0.62) for non-prediabetic.

## Discussion

This cohort study yields several significant findings: (1) Participants recovered from dyslipidemia still face an elevated risk of T2D, surpassing even that of the normal-developed dyslipidemia group. (2) In this specific population, nontraditional lipid parameters such as LCI, AIP, NHDL, and TyG, along with the traditional parameter TG, exhibit strong and consistent associations with increased T2D risk across both continuous and categorical analyses. (3) In non-prediabetic individuals, LCI, AIP, TyG, and TG remain significantly associated with T2D risk, highlighting their utility in identifying latent metabolic risks. (4) Among all traditional and nontraditional lipid parameters, TyG has the strongest capacity to identify both the overall and prediabetic populations, while AIP shows the best C-index for identifying non-prediabetic individuals.

These findings underscore crucial implications for public health and clinical practice. The findings indicate that individuals who achieve normalization of lipid levels have a significantly reduced relative risk of T2D compared to those with persistent dyslipidemia, underscoring the critical role of timely intervention in lipid abnormalities for improving lipids metabolic health. However, we observed that even after lipid levels return to normal, the risk of T2D remains in this group of individuals with both blood glucose and lipid levels within the normal range. In clinical reception, it is essential not only to focus on the current glucose and lipids levels but also to give additional attention to individuals with a history of dyslipidemia. The long-term impact of prior lipid abnormalities cannot be overlooked, highlighting the urgent need for more refined and individualized monitoring and prevention of T2D risk.

Based on this, our study further demonstrates that among the nontraditional lipid parameters, the LCI, AIP and TyG index are significantly associated with an elevated T2D risk in this population. Moreover, in non-prediabetic individuals, these indices may reveal underlying metabolic disturbances before glucose abnormalities become apparent. Therefore, in clinical practice and large-scale screening, a comprehensive assessment of glucose levels and nontraditional lipid parameters may provide a more complete picture of an individual’s T2D risk, in order to shift the focus of T2D prevention earlier.

Current research has explored the impact of lipid metabolism disorders on T2D. Several plasma lipidomics studies have demonstrated that lipid levels undergo significant changes years before the onset of T2D or prediabetes, monitoring relevant lipid parameters can facilitate the early identification of high-risk individuals who may benefit from timely interventions (24, 25). However, the high cost and technical complexity of omics measurements limit their application in large-scale screening. In recent years, nontraditional lipid parameters (such as AIP, TyG, etc.…) derived from traditional lipid parameters have been recognized for their superior clinical utility in reflecting the degree of insulin resistance and assessing the risk of T2D.

However, research that comprehensively compares nontraditional indicators using large-scale longitudinal cohorts remains relatively scarce. A cohort study based on an Iranian population found that AIP and NHDL, independent of traditional risk factors, were associated with the risk of T2D, but the comparison was mainly made with traditional lipid parameters (26). Another cross-sectional study based on a Chinese population revealed associations between several nontraditional lipid parameters, including LCI, AIP, CRI-I, and NHDL, and prediabetes (7). Similarly, a cohort study involving a Japanese population without T2D identified that the TG/HDL ratio and CRI-I were also associated with an increased risk of T2D, consistent with our findings (27). Furthermore, several cross-sectional studies based on the National Health and Nutrition Examination Survey suggested that AIP (28–30) and RC (29–31) are significantly associated with diabetes, and insulin resistance may play a mediating role (29). In these studies, nontraditional lipid parameters demonstrated significant associations with T2D or prediabetes, underscoring their potential role in metabolic risk.

Our study emphasizes a group whose lipid profiles have ostensibly returned to normal, but still considered at high risk for T2D. Several parameters, particularly AIP, demonstrated a strong ability to capture subtle metabolic disturbances, which is consistent with the findings of existing studies (29, 30, 32). Building on this, our study further found that AIP can still capture the elevated risk of diabetes even after lipid levels have returned to normal. In contrast, traditional lipid parameters like HDL and LDL were no longer significantly associated with T2D risk. Although TG remained associated with T2D, the levels within the normal range could easily lead clinicians to underestimate individual’s risk. This highlights the limitations of relying solely on traditional lipid metrics (33), which may underestimate lingering metabolic dysregulation. However, our findings only suggested a potential positive effect between RC and RHC and T2D, implicating that they may not be the most effective parameters for detailed risk screening within this subgroup. Furthermore, compared to previous studies, we included TyG in our comparisons, providing additional evidence supporting its utility in T2D prevention. Besides, compared to other studies, this research conducted multiple fasting glucose measurements through regular follow-ups, which is more likely to avoid potential healthy selection bias and more accurately reflect the true incidence of T2D.

Even after dyslipidemia is restored, the risk of T2D remains elevated, possibly due to the irreversible damage caused by lipid-mediated apoptosis to pancreatic β-cells (34, 35). Prolonged exposure to elevated fatty acid metabolites enhances apoptosis (3), leading to pancreatic dysfunction and elevated T2D risk (36). Furthermore, elevated plasma TG, fatty acids, and LDL can induce renal dysfunction by promoting lipotoxicity, insulin resistance, and pro-inflammatory pathways (37), including damage to proximal tubule cells (38) and the development of glomerulosclerosis (39), which is considered as an irreversible process and will exacerbate insulin resistance through various mechanisms (40). Furthermore, lipid metabolism disorders may also cause persistent reprogramming of the innate immune system, which can endure long after metabolic abnormalities are normalized (41).

The strengths of our study include its longitudinal design, long follow-up duration, stable follow-up population, and comprehensive comparison of nontraditional and traditional lipid parameters, as well as being the first to focus on individuals recovering from dyslipidemia. However, this study also has some limitations, including the inability of observational studies to infer causality, the relatively small sample size for specific subgroups, and the fact that participants were mainly from northern China, which may limit the generalizability of the findings. Additionally, we were unable to differentiate between T2D and type 1 diabetes (T1D). However, the impact of misclassifying T1D was minimal, as T2D accounts for approximately 95% of all diabetes cases. Moreover, the average age of participants at follow-up (51.9 years) exceeds the typical age of onset for T1D. Another limitation is that, due to the lack of available genetic data in the Kailuan cohort, we were unable to evaluate potential gene–environment interactions. Future large-scale, multicenter studies with diverse populations and integrated genetic information are needed to further investigate the long-term metabolic outcomes and underlying mechanisms among individuals recovering from dyslipidemia. In addition, the associations of these nontraditional lipid parameters with diabetic complications and the potential specificity of these associations also require further investigation. In conclusion, our study expands the understanding of nontraditional lipid parameters, particularly in populations that appear normolipidemic yet remain at high risk for T2D. The research provides theoretical support for the clinical application of nontraditional lipid parameters as parameters for early intervention and precision medicine in T2D care.

## Data Availability

The data analyzed in this study is subject to the following licenses/restrictions: The original data supporting the findings of this study are available from the corresponding author upon reasonable request. Requests to access these datasets should be directed to Dr Shouling Wu, drwusl@163.com.
