# Supplementary material for: Relationship between nontraditional lipid parameters and the risk of type 2 diabetes in individuals recovered from dyslipidemia: a cohort study
Source: Front Endocrinol (Lausanne). 2025 Sep 25;16:1610091. doi: 10.3389/fendo.2025.1610091 (PMC12507547; doi:10.3389/fendo.2025.1610091)
Supplement: Supplementary file 1 [file Table1.docx]

| **Table S1. Missing covariates** | | |
| --- | --- | --- |
| **Covariate** | **Number of Missing Values** | **Percentage of Missing Values** |
| Age | 0 | 0.00% |
| Sex | 0 | 0.00% |
| Systolic blood pressure | 775 | 1.97% |
| Body mass index | 2,033 | 5.18% |
| Estimated glomerular filtration rate | 79 | 0.20% |
| Smoking | 0 | 0.00% |
| Alcohol consumption | 0 | 0.00% |
| Physical activity | 144 | 0.37% |
| Education level | 1 | <0.01% |
| Use of antihypertensive medications | 103 | 0.26% |

| **Table S2. 2-year lagged analysis** | | | | |  |
| --- | --- | --- | --- | --- | --- |
|  | **Persistent**  **normal** | **Dyslipidemia-**  **recovered** | **Normal-**  **developed** | **Persistent Dyslipidemia** | **P value** |
| Events/Total | 1,695/18,209 | 527/3,818 | 1,120/8,736 | 1,572/8,296 |  |
| IR, per 1000 person-years | 11.14 | 17.03 | 15.85 | 24.08 |  |
| Model 1 | 1（reference） | 1.51 (1.37,1.66) | 1.46 (1.36, 1.58) | 2.18 (2.03, 2.33) | <0.001 |
| Model 2 | 1（reference） | 1.36 (1.23, 1.50) | 1.31 (1.21, 1.41) | 1.74 (1.62, 1.87) | 0.001 |
| Model 1 was adjusted for age and sex. | | | | |  |
| Model 2 was further adjusted for systolic blood pressure, estimated glomerular filtration rate, body mass index, smoking, alcohol consumption, physical activity, education level, and the use of antihypertensive medications. | | | | |  |
| HR, hazard ratio. IR, incidence rate. | | | | |  |

| **Table S3. Fine-Gray model for competition** | | | | |  |
| --- | --- | --- | --- | --- | --- |
|  | **Persistent**  **normal** | **Dyslipidemia-**  **recovered** | **Normal-**  **developed** | **Persistent**  **Dyslipidemia** | **P value** |
| Events/Total | 3,418/18,209 | 931/3,850 | 1,931/8,803 | 2,549/8,421 |  |
| IR, per 1000 person-years | 22.44 | 30.03 | 27.28 | 38.91 |  |
| Model 1 | 1（reference） | 1.52 (1.38, 1.67) | 1.47 (1.36, 1.58) | 2.22 (2.07, 2.37) | <0.001 |
| Model 2 | 1（reference） | 1.37 (1.25, 1.51) | 1.31 (1.21, 1.41) | 1.78 (1.66, 1.91) | <0.001 |
| Model 1 was adjusted for age and sex. | | | | |  |
| Model 2 was further adjusted for systolic blood pressure, estimated glomerular filtration rate, body mass index, smoking, alcohol consumption, physical activity, education level, and the use of antihypertensive medications. | | | | |  |
| HR, hazard ratio. IR, incidence rate. | | | | |  |

| **Table S4. Excluded participants receiving antihypertensive drug treatment** | | | | |  |
| --- | --- | --- | --- | --- | --- |
|  | **Persistent**  **normal** | **Dyslipidemia-**  **recovered** | **Normal-**  **developed** | **Persistent Dyslipidemia** | **P value** |
| Events/Total | 1,589/16,864 | 486/3,462 | 1,012/7,814 | 1,366/6,974 |  |
| IR, per 1000 person-years | 11.19 | 17.26 | 15.93 | 24.85 |  |
| Model 1 | 1（reference） | 1.51 (1.37, 1.68) | 1.46 (1.35, 1.58) | 2.23 (2.07, 2.40) | <0.001 |
| Model 2 | 1（reference） | 1.36 (1.23, 1.51) | 1.32 (1.22, 1.44) | 1.84 (1.71, 1.98) | <0.001 |
| Model 1 was adjusted for age and sex. | | | | |  |
| Model 2 was further adjusted for systolic blood pressure, estimated glomerular filtration rate, body mass index, smoking, alcohol consumption, physical activity, education level, and the use of antihypertensive medications. | | | | |  |
| HR, hazard ratio. IR, incidence rate. | | | | |  |

| **Table S5. Excluded participants receiving lipid-lowering drug treatment** | | | | |  |
| --- | --- | --- | --- | --- | --- |
|  | **Persistent**  **normal** | **Dyslipidemia-**  **recovered** | **Normal-**  **developed** | **Persistent Dyslipidemia** | **P value** |
| Events/Total | 1,780/18209 | 559/3,850 | 1,169/8,685 | 1,652/8,216 |  |
| IR, per 1000 person-years | 11.69 | 18.03 | 16.73 | 25.85 |  |
| Model 1 | 1（reference） | 1.52 (1.38, 1.67) | 1.47 (1.36, 1.58) | 2.22 (2.08, 2.38) | <0.001 |
| Model 2 | 1（reference） | 1.37 (1.25, 1.51) | 1.32 (1.22, 1.42) | 1.78 (1.66, 1.91) | <0.001 |
| Model 1 was adjusted for age and sex. | | | | |  |
| Model 2 was further adjusted for systolic blood pressure, estimated glomerular filtration rate, body mass index, smoking, alcohol consumption, physical activity, education level, and the use of antihypertensive medications. | | | | |  |
| HR, hazard ratio. IR, incidence rate. | | | | |  |

| **Table S6. Additional Adjustment for Baseline Fasting Blood Glucose** | | | | |  |
| --- | --- | --- | --- | --- | --- |
|  | **Persistent**  **normal** | **Dyslipidemia-**  **recovered** | **Normal-**  **developed** | **Persistent Dyslipidemia** | **P value** |
| Events/Total | 1,780/18,209 | 559/3,850 | 1,187/8,803 | 1,697/8,421 |  |
| IR, per 1000 person-years | 11.69 | 18.03 | 16.73 | 25.85 |  |
| Model 1 | 1（reference） | 1.52 (1.38, 1.67) | 1.47 (1.36, 1.58) | 2.22 (2.08, 2.38) | <0.001 |
| Model 2 | 1（reference） | 1.29 (1.18, 1.42) | 1.21 (1.13, 1.31) | 1.54 (1.44, 1.65) | <0.001 |
| Model 1 was adjusted for age and sex. | | | | |  |
| Model 2 was further adjusted for systolic blood pressure, estimated glomerular filtration rate, body mass index, smoking, alcohol consumption, physical activity, education level, the use of antihypertensive medications and fasting blood glucose. | | | | |  |
| HR, hazard ratio. IR, incidence rate. | | | | |  |

| **Table S7. Relationship between novel and traditional lipid parameters and type 2 diabetes,** | | | | | | | |
| --- | --- | --- | --- | --- | --- | --- | --- |
|  | **Q1** | **Q2** | **Q3** | **Q4** | **P for overall** | **Continuous variables** | **P value** |
| LCI | Reference | 1.67 (1.27, 2.22) | 1.66 (1.26, 2.20) | 2.10 (1.61, 2.75) | <0.001 | 1.03 (1.02, 1.04) | 0.001 |
| AIP | Reference | 1.31 (0.99, 1.73) | 1.47 (1.12, 1.93) | 1.97 (1.51, 2.55) | <0.001 | 1.87 (1.52, 2.28) | <0.001 |
| NHDL | Reference | 1.11 (0.85, 1.45) | 1.43 (1.11, 1.83) | 1.39 (1.08, 1.79) | 0.013 | 1.21 (1.06, 1.38) | <0.001 |
| CRI-I | Reference | 1.15 (0.88, 1.50) | 1.34 (1.03, 1.75) | 1.38 (1.06, 1.79) | <0.001 | 1.23 (1.09, 1.38) | <0.001 |
| CRI-II | Reference | 1.34 (1.03, 1.75) | 1.38 (1.07, 1.80) | 1.37 (1.06, 1.78) | <0.001 | 1.21 (1.05, 1.41) | <0.001 |
| RC | Reference | 1.31 (1.02, 1.69) | 1.26 (0.98, 1.63) | 1.38 (1.07, 1.77) | 0.068 | 1.09 (0.96, 1.24) | 0.183 |
| RHC | Reference | 1.11 (0.86, 1.43) | 1.22 (0.95, 1.57) | 1.33 (1.04, 1.70) | 0.132 | 1.15 (0.98, 1.35) | 0.094 |
| TyG | Reference | 1.66 (1.23, 2.24) | 1.91 (1.42, 2.56) | 3.24 (2.46, 4.26) | <0.001 | 3.70 (2.88, 4.75) | <0.001 |
| HDL | Reference | 1.02 (0.82, 1.27) | 0.92 (0.73, 1.17) | 0.76 (0.58, 1.00) | 0.136 | 0.68 (0.51, 0.91) | <0.001 |
| TG | Reference | 1.32 (1.00, 1.73) | 1.46 (1.12, 1.91) | 1.96 (1.53, 2.53) | <0.001 | 1.87 (1.54, 2.26) | <0.001 |
| LDL | Reference | 1.34 (1.05, 1.72) | 1.13 (0.87, 1.46) | 1.25 (0.98, 1.60) | 0.110 | 1.10 (0.97, 1.25) | 0.137 |
| Relationship between novel and traditional lipid parameters and type 2 diabetes, analyzed as both quartiles and continuous variables | | | | | | | |

| **Table S8. The quartile cut-off values for lipid parameters** | | | | |
| --- | --- | --- | --- | --- |
|  | **First quartile** | **Second quartile** | **Third quartile** | **Fourth quartile** |
| LCI | 0<LCI≤6.40 | 6.40<LCI≤10.18 | 10.18<LCI≤14.69 | LCI＞14.69 |
| AIP | -2.57<AIP≤-0.51 | -0.51<AIP≤-0.17 | -0.17<AIP≤0.12 | AIP＞0.12 |
| NHDL | 0.68<NHDL≤2.92 | 2.92<NHDL≤3.40 | 3.40<NHDL≤3.86 | NHDL＞3.86 |
| CRI-I | 0.38<CRI-I≤1.83 | 1.83<CRI-I≤2.33 | 2.33<CRI-I≤2.91 | CRI-I＞2.91 |
| CRI-II | 0.14<CRI-II≤1.37 | 1.37<CRI-II≤1.75 | 1.75<CRI-II≤2.09 | CRI-II＞2.09 |
| RC | 0.01<RC≤0.39 | 0.39<RC≤0.71 | 0.71<RC≤1.20 | RC＞1.20 |
| RHC | 0.01<RHC≤0.25 | 0.25<RHC≤0.47 | 0.47<RHC≤0.86 | RHC＞0.86 |
| TyG | 6.36<TyG≤8.25 | 8.25<TyG≤8.54 | 8.54<TyG≤8.79 | TyG＞8.79 |
| HDL | 1.03<HDL≤1.24 | 1.24<HDL≤1.42 | 1.42<HDL≤1.68 | HDL＞1.68 |
| TG | 0.16<TG≤0.93 | 0.93<TG≤1.22 | 1.22<TG≤1.53 | TG＞1.53 |
| LDL | 0.21<LDL≤2.13 | 2.13<LDL≤2.50 | 2.50<LDL≤2.93 | LDL＞2.93 |

| **Table S9 . Novel and traditional lipid parameters for prediabetes stratification in Cox regression analysis, with each 1-SD change.** | | | | | | |
| --- | --- | --- | --- | --- | --- | --- |
|  | **Total** | **P value** | **Prediabetes** | **P value** | **Non-prediabetes** | **P value** |
| LCI | 1.22 (1.14, 1.32) | <0.001 | 1.21 (1.09, 1.34) | <0.001 | 1.13 (1.00, 1.28) | 0.047 |
| AIP | 1.35 (1.22, 1.49) | <0.001 | 1.37 (1.21, 1.56) | <0.001 | 1.25 (1.07, 1.44) | <0.001 |
| NHDL | 1.13 (1.04, 1.24) | <0.001 | 1.14 (1.01, 1.28) | 0.033 | 1.07 (0.93, 1.22) | 0.364 |
| CRI-I | 1.16 (1.06, 1.27) | <0.001 | 1.17 (1.05, 1.32) | <0.001 | 1.10 (0.96, 1.27) | 0.167 |
| CRI-II | 1.12 (1.02, 1.21) | 0.011 | 1.12 (1.00, 1.26) | 0.051 | 1.00 (0.87, 1.15) | 0.988 |
| RC | 1.06 (0.97, 1.14) | 0.182 | 1.07 (0.96, 1.19) | 0.215 | 1.12 (0.99, 1.26) | 0.081 |
| RHC | 1.07 (0.99, 1.16) | 0.085 | 1.08 (0.98, 1.20) | 0.130 | 1.13 (0.99, 1.27) | 0.051 |
| TyG | 1.71 (1.54, 1.89) | <0.001 | 1.50 (1.32, 1.71) | <0.001 | 1.28 (1.11, 1.48) | <0.001 |
| HDL | 0.87 (0.79, 0.97) | 0.014 | 0.83 (0.73, 0.95) | <0.001 | 0.91 (0.77, 1.07) | 0.232 |
| TG | 1.31 (1.21, 1.43) | <0.001 | 1.33 (1.19, 1.49) | <0.001 | 1.22 (1.07, 1.39) | <0.001 |
| LDL | 1.07 (0.98, 1.16) | 0.140 | 1.05 (0.94, 1.18) | 0.382 | 0.95 (0.83, 1.08) | 0.446 |
| Abbreviation: SD, standard deviation. | | | | | | |

| **Table S10. The C-index for lipid parameters and fasting blood glucose** | | | |
| --- | --- | --- | --- |
|  | **Total** | **Prediabetes** | **Non-prediabetes** |
| LCI | 0.59 (0.57, 0.62) | 0.58 (0.55, 0.61) | 0.57 (0.54, 0.61) |
| AIP | 0.59 (0.57, 0.62) | 0.59 (0.56, 0.62) | 0.58 (0.55, 0.62) |
| NHDL | 0.56 (0.54, 0.59) | 0.56 (0.53, 0.60) | 0.55 (0.51, 0.58) |
| CRI-I | 0.57 (0.54, 0.59) | 0.57 (0.53, 0.60) | 0.57 (0.53, 0.60) |
| CRI-II | 0.56 (0.53, 0.58) | 0.55 (0.51, 0.58) | 0.55 (0.52, 0.58) |
| RC | 0.53 (0.50, 0.55) | 0.54 (0.51, 0.58) | 0.55 (0.51, 0.59) |
| RHC | 0.54 (0.51, 0.56) | 0.55 (0.52, 0.59) | 0.56 (0.52, 0.60) |
| TyG | 0.64 (0.62, 0.67) | 0.62 (0.58, 0.65) | 0.57 (0.54, 0.61) |
| HDL | 0.54 (0.51, 0.56) | 0.54 (0.51, 0.57) | 0.56 (0.52, 0.59) |
| TG | 0.59 (0.57, 0.62) | 0.59 (0.56, 0.62) | 0.57 (0.53, 0.60) |
| LDL | 0.54 (0.52, 0.57) | 0.53 (0.49, 0.56) | 0.51 (0.48, 0.54) |
| The C-index values are displayed as point estimates with their 95% confidence intervals. | | | |
